# Medical Characteristics and Therapeutic Approaches Used to Treat Primary and Secondary Infertile Women

**Published:** 2018-12

**Authors:** Abdelhafid BENKSIM, Rachid AIT ADDI, Noureddine ELKHOUDRI, Mohamed CHERKAOUI

**Affiliations:** 1. Laboratory of Human Ecology, Dept. of Biology, School of Sciences Semlalia, Cadi Ayyad University, Marrakech. Morocco; 2. Laboratory of Biology, High Institute of Nursing and Technical of Health, Marrakesh, Moro; 3. Laboratory of Sciences and Health Technologies, Higher Institute of Health Sciences University Hassan First, Settat, Morocco

**Keywords:** Women, Fertility drugs, Assisted reproductive treatments

## Abstract

**Background:**

Inability to conceive is a major problem during reproductive age. This study aimed to describe medical characteristics and different approaches to get better the management of infertility among women referring to some public and private health centers in Morocco.

**Methods:**

Overall, 619 infertile women referring to public and private health centers in Marrakech-Safi region were selected by simple random sampling method, between 1 Oct 2013 and 31 Dec 2015. The socio-economic data, demographic characteristics, medical and obstetric variables and types of infertility treatments were simultaneously collected by questionnaire and health record data. The univariate logistic regression analyses were used to determine different infertility treatments. Statistical significance was set at 0.05.

**Results:**

The rate of primary and secondary infertility was 67.37%, and 32.63%, respectively. In comparison to secondary infertility, primary infertile women with high socio-economic level and low average age have used many fertility drugs and assisted reproductive technologies (Clomifene citrate (45.01 *vs.* 29.20%), injectable gonadotropins (09.35 *vs.* 3.96%), dydrogesterone (35.08 *vs.* 23.26%), intrauterine insemination (3.83 vs. 0.49%), and in-vitro fertilisation (3.11 *vs.* 1.48%)).

**Conclusion:**

The use of infertility’s treatment is limited in Morocco. Outside of medical coverage, the infertility management requires permanent efforts, financial supports, psychological assistance and serious dialogue between all the stakeholders.

## Introduction

Infertility is defined as the failure of conception after at least 12 months of unprotected intercourse (1,2). It is a public health problem in reproductive age affecting more than 10% of the world’s population and about 12% in Moroccan population (1–3). A number of factors can dislocate the process of fertility at any step. There were several causes of infertility including couple’s age, menstrual disorders, tubal dysfunction, uteri-cervical anomaly, environmental pollutants and sperm abnormalities (2, 4–6). Although some couples use only one or two therapies to achieve pregnancy.

The clinical care provider may suggest a specific treatment if the causes of infertility have been identified. However, when traditional treatments are failed, fertility drugs are often the first steps in infertility treatment. They are regularly used for assisted reproductive technologies (ART) (7). These treatments may include drugs for ovarian stimulation, oocyte maturation and premature ovulation (7). Moreover, assisted reproductive treatment for infertile couples now leads to reasonably high pregnancy rates. These pregnancies are often associated with the risk of ovarian hyperstimulation syndrome or multiple pregnancies (8). This outcome requires the adoption of proven and personalized diagnostic and therapeutic approaches to optimize efficacy and safety outcomes (9).

Firstly, clomiphene citrate is used in superovulation by causing the pituitary gland to release more FSH and LH, which stimulate the growth of an ovarian follicle containing eggs (10–11). Moreover, gonadotropins are hormones (LH and FSH) given in an injection to stimulate a woman’s ovaries to produce follicles, which contain an egg (12). Thus, dydrogesterone can be used to treat a lack of progesterone including menstrual disorders, spontaneous abortion, endometriosis and infertility (13). In the end, the assisted reproductive technologies (ART) included intrauterine insemination (IUI) (4), in vitro fertilization, and intracytoplasmic sperm injection (13–15).

Few studies were dedicated to infertility treatment in Morocco. Indeed, our aim was to describe the medical characteristics and the different types of infertility treatment in women referred to five public and private health centers in Morocco.

## Materials and Methods

### Design study

Ethical approval was obtained from the authorities of health in region Marrakech-Safi. This region is located in the middle of Morocco and consists of the one prefecture and eight administrative provinces. Our interest in this cross-sectional study was to describe several approaches used to treat primary and secondary infertile women and deduct some factors associated. This study was conducted at different public and private health centers in the region of Marrakech-Safi (Zerktouni, Youssef Ben Tachefine, Ibn Zohr, Mohamed Ben Abdellah, and Ibn Tofail hospital). Indeed, 619 infertile women referring to these health centers was recruited in medical consultations (general practitioners or obstetrician/gynaecologists) by simple random sampling method, between 1 Oct 2013 and 31 Dec 2015 without previously appointment. Subsequently, on average, 123 women were selected at each health center for 619 infertile women. The study protocol was explained and the informed consent obtained from infertile women before enrolment. All data were simultaneously collected by questionnaire and health record data of each married woman. These two tolls provided different data: socioeconomic data, demographic characteristics, age of couple, medical and obstetric history, hormone levels, semen analysis, methods of diagnosis and different types of treatment approaches have been recorded. All data were collected by qualified nursing students for a better understanding.

Exclusion criteria include single and post-menopausal women. Inclusion criteria include married women of childbearing age who had obstacles to become a mother after at least 12 months of regular sexual intercourse.

### Statistical analyses

Data were statistically analyzed using SPSS for Windows ver. 10.0 (Chicago, IL, USA) software. All experimental data were expressed as mean ± standard deviation (SD) or number (percentage). Chi-square test and exact test of Fisher were used for categorical variables. Student’s t-test was used to estimate the observed differences between the means. The *P*<0.05 level of probability was used as the criteria of significance for the two tails.

## Results

### Description of study

Overall, 619 infertile women were included in this study whose 417 (67.37%) with primary and 202 (32.63%) with secondary infertility (Table 1). The mean women’s age was 28.7 yr (SD±5.7) in primary and 31.95 yr (SD± 5.6) in secondary infertility (*P*=0.001).

**Table 1:** Socioeconomic and medical characteristics of infertile women

***Variables and modalities***	***Primary infertility n= 417***	***Secondary infertility n= 202***	***Χ^2^/t-test***
Woman’s age (year) **^a^**	28.7 ± 5.7	31.95 ± 5.6	45.76^***^
Husband’s age (year) **^a^**	35.8 ± 7.7	38.8 ± 6.8	39.07^***^
Socioeconomic level of couple **^b^**			
Low level	28 (6.70)	26 (12.90)	10.91^**^
Average level	389 (93.30)	176 (87.10)	
Duration of infertility: > 3.8 yr **^b^**	148 (35.5)	90 (44.6)	ns
Menstrual disorders **^b^**	213 (51,1)	92 (45.5)	ns
Tubal dysfunction **^b^**	105 (64.40)	41 (68.30)	ns
Uterine and cervical anomalies **^b^**	65 (15.6)	33 (16.32)	ns
Spontaneous abortion	00 (00)	71 (30.14)	21.59^***^
Husband’s consultation **^b^**	239 (57.3)	60 (29.70)	36.91^***^
Anomaly of sperm **^b^**	107 (45.10)	12 (20.31)	26.01^***^
Frequency of intercourse: **^b^**			
< 3 times per week	166 (39.80)	55 (27.22)	ns

Mean ± SD or Number (%);

a Student’s *t*-test;

b Chi-squared test.

ns: non significant;

* *P*<0.05;

** *P*<0.01;

*** *P*<0.001

The average age of husbands was 35.8 yr (±7.7) and 38.8 yr (±6.8) for primary and secondary infertility, respectively (*P*=0.001). Moreover, an elevated socio-economic situation was reported in primary infertility than secondary infertility (93.3% vs. 87.1%) (*P*=0.003). According to infertile women, the average duration of infertility was 3.8 ± 3.6 yr. Hence, the long duration of infertility was reported in secondary infertility than primary infertility (44.6% vs. 35.5%) (*P*=0.075).

In comparison to primary infertility, the principal causes of secondary infertility were relatively due to tubal dysfunction (64.40% vs. 68.30%), spontaneous abortion (30.14%), uterine and cervical anomalies (6.9% vs. 8.2%) without significant difference. However, the main causes of primary infertility were due to menstrual disorders (51.1% vs. 45.5%), and abnormalities of sperm (45.1% vs. 20.3%) with significant differences. In addition, 70.30% of secondary infertile men have unfortunately refused to see an infertility specialist and only 42.70% of them in primary infertility (*P*=0.001). Furthermore, if the couple want to increase their chance to conceive, some researchers have recommended regular sexual intercourse (2–3 times per week) after menstruation and that when the woman does not know exactly the day of her ovulation (16,17). In this study, the frequency of sexual intercourse in secondary infertility was far higher than primary infertility (39.80% vs. 27.22%) (*P*=0.003).

All therapeutic approaches were presented in Table 2. In comparison to secondary infertility, clomiphene citrate was taken as initial treatment to induce ovulation in primary infertile women (45.01 vs. 29.20%) (*P=*0.003). In addition, gonadotropins hormones can be given as intramuscular or subcutaneous injections (9.35 vs. 3.96%) (*P=*0.674). Dydrogesterone is also taken to treat primary than secondary infertility (35.08 vs. 23.26%) (*P*=0.002). Furthermore surgeries were slightly practiced for treatment of primary and secondary infertility (<8.65%). According to infertile women, if fertility drugs were failed, IUI is often practiced in primary than secondary infertility (3.83% vs. 0.49%) without significant difference. Moreover, in vitro-fertilisation (IVF) is practiced by couples who were never able to conceive before (3.11%) and only by 1.48% in secondary infertility (*P*=0.091).

**Table 2:** Different types of fertility drugs used to treat infertile women

***Variables and modalities***	***Primary infertility N = 417***	***Secondary infertility N = 202***	***Χ^2^***
Clomifene citrate 50mg **^b^**	188 (45.08)	59 (29.20)	10.91^**^
Oral dydrogesterone 10mg **^b^**	146 (35.01)	47 (23.26)	10.61^**^
Gynaecological surgery **^b^**	45 (10.2)	15 (7.10)	ns
Injectable gonadotropins (FSH, LH) **^b^**	39 (09.35)	08 (3.96)	ns
Intrauterine insemination (IUI) **^b^**	16 (3.83)	01 (0.49)	ns
In-vitro fertilisation (IVF) **^b^**	13 (3.11)	03 (1.48)	ns

Mean ± SD or Number (%);

a Student’s t-test;

b Chi-squared test.

ns: non-significant;

* *P*<0.05;

** *P*<0.01;

*** *P*<0.001

## Discussion

To our knowledge, this is a first study showing the medical characteristics and several approaches to treat primary and secondary infertility in Morocco. In our country, few studies have been devoted to treatment of infertility. The age of women is considered a prognostic factor when the type of fertility treatment is proposed. The high age of women can reduce the chance of pregnancy among women who were under treatment (2, 5, 18). In addition, there was a relationship between woman’s age and type of infertility treatment (5, 6).

The success rate of assisted reproductive technologies began to decline from age 35 and particularly from age 40 (5,6). Furthermore, the age of woman in secondary infertility was an obstacle to achieve a natural pregnancy. However, the age of women with primary infertility can give them another opportunities to try different types of fertility treatment (*P*=0.001). In comparison with secondary infertility, the high socioeconomic status of primary infertile women (93.3%) allows them to test several programs of fertility including, clomifene citrate (45.01%), gonadotrophins (09.35%), dydrogesterone (35.08%), intra-uterine insemination (3.83%) and in vitro fertilization (3.11%). Socioeconomic level is relatively associated to chosen approach and will explain the limited access to sophisticated reproductive technologies (13). Overall, 56% of people seek medical treatment for infertility, but only 22.4% benefit from this treatment (3).

However, despite the high rate of tubal dysfunction (64.40% to 68.30%), sperm abnormalities (20.31% to 45.10%) and irregular frequency of intercourse (27.22% to 39.80%), fertility drugs remain the first line therapy among many infertile women. Thus, clomiphene citrate is slightly taken in secondary infertility (11). Despite their advanced age many secondary infertile women who have always attempted to achieve a pregnancy naturally without superovulation.

Primary infertility is diagnosed and treated much more than secondary infertility (2). Although clomiphene citrate induces ovulation at 73% of cases and the occurrence of pregnancy does not exceed 36% (7). On the other hand, oral dydrogesterone was greatly taken to treat some causes of primary infertility (35.08%). This drug is taken in the late luteal phase might have a potential benefit for infertility associated with irregular menstruation (13). Overall, the problem of tubal dysfunction and partner’s consultation is relatively dropping the success of fertility drugs, assistance reproductive technology, and the chance of pregnancy.

Moreover, sexual disorders related to duration of medical therapy are common among infertile couples (19). Moreover, there was a higher prevalence of sexual dysfunction in secondary infertile women (19). In this study, the low frequency of sexual intercourse was often due to lifestyle associated with frequent compulsory travels requiring staying away from home or to sexual problems in one or both (20). When pregnancy does not occur, the couples think that sexual intercourse is not fruitful, and sexual desire can be reduce (17). Therefore, it was necessary to help infertile couples for rebuilding their sexuality with a lot of mutual pleasure, knowing that in some cultures infertility is considered a taboo and many people were ashamed to talk about it, which reduces the effectiveness of diagnosis and treatment (ART) (1). In addition, injectable gonadotropins were uncomfortable, unpleasant and expensive, which may be explain their low uses (20). Furthermore, surgical treatments were slightly practiced. It is may be due to success of fertility treatments and infertile women often start with less invasive medical procedures (1).

Many infertile couples were not able to practice assisted reproductive treatments, because these technologies were expensive, complex and sometimes stressful without effective medical coverage. Some studies showed that anxiety and depression symptoms were observed in 14.7% to 17.9% of cases (17,18).

## Conclusion

The treatment of infertility is not easy to understand due to intricacy of medical protocols. There were a lot of treatments available, which will depend on the causes of infertility. Effective treatment requires accurate assessment of the underlying cause of infertility. In general, a good collaboration between psychologists, infertility specialists and sexologists would certainly help these spouses to preserve a better sexual life and probably treat problems related to infertility.

## Ethical considerations

Ethical issues (Including plagiarism, informed consent, misconduct, data fabrication and/or falsification, double publication and/or submission, redundancy, etc.) have been completely observed by the authors.
